# Spin caloritronic nano-oscillator

**DOI:** 10.1038/s41467-017-00184-5

**Published:** 2017-07-18

**Authors:** C. Safranski, I. Barsukov, H. K. Lee, T. Schneider, A. A. Jara, A. Smith, H. Chang, K. Lenz, J. Lindner, Y. Tserkovnyak, M. Wu, I. N. Krivorotov

**Affiliations:** 1 Department of Physics and Astronomy, University of California, Irvine, California 92697 USA; 2 Helmholtz-Zentrum Dresden–Rossendorf, Institute of Ion Beam Physics and Materials Research, Bautzner Landstrasse 400, 01328 Dresden, Germany; 3 Department of Physics, Colorado State University, Fort Collins, Colorado 80523 USA; 4 Department of Physics and Astronomy, University of California, Los Angeles, California 90095 USA

## Abstract

Energy loss due to ohmic heating is a major bottleneck limiting down-scaling and speed of nano-electronic devices, and harvesting ohmic heat for signal processing is a major challenge in modern electronics. Here, we demonstrate that thermal gradients arising from ohmic heating can be utilized for excitation of coherent auto-oscillations of magnetization and for generation of tunable microwave signals. The heat-driven dynamics is observed in Y_3_Fe_5_O_12_/Pt bilayer nanowires where ohmic heating of the Pt layer results in injection of pure spin current into the Y_3_Fe_5_O_12_ layer. This leads to excitation of auto-oscillations of the Y_3_Fe_5_O_12_ magnetization and generation of coherent microwave radiation. Our work paves the way towards spin caloritronic devices for microwave and magnonic applications.

## Abstract (web summary)

Harvesting ohmic heat for signal processing is one of major challenges in modern electronics and spin caloritronics, but not yet well accomplished. Here the authors demonstrate a spin torque oscillator device driven by pure spin current arising from thermal gradient across an Y_3_Fe_5_O_12_/Pt interface.

## Introduction

Nano-devices based on control of magnetic damping by spin currents^1, 2^, such as spin torque memory and spin torque oscillators^3–7^ (STOs), are at the forefront of spintronics research. In STOs, a spin current injected into a ferromagnet acts as negative magnetic damping that cancels the positive damping of magnetization at a critical current density^3–12^. Above the critical current, magnetization auto-oscillations are excited with an amplitude determined by nonlinearities of the magnetic system^13^. By utilizing magnetoresistive effects, the auto-oscillations can be converted into microwave power. STOs can also serve as sources of propagating spin waves for spin wave logic and nano-magnonic applications^14, 15^. STOs are typically metallic devices that rely either on spin-polarized electric currents^3–5^ or on pure spin currents generated by the spin Hall effect^7–9, 11, 12, 16^. Recently, an STO based on injection of pure spin Hall current into an insulating ferromagnet was demonstrated as well^7^.

Spin angular momentum can be transferred from a non-magnetic metal (N) to a ferromagnetic insulator (F) via inelastic spin flip scattering of a conduction electron at the F/N interface that generates a magnon in the F layer^15, 17–20^. The inverse process is responsible for the spin pumping effect whereby angular momentum of magnons in the F layer is converted into spin accumulation that drives spin current in the N layer^21, 22^. At a non-zero temperature, these two processes give rise to a fluctuating spin current flowing across the F/N interface that time-averages to zero in thermal equilibrium^21, 23^. When a thermal gradient is applied perpendicular to the F/N interface, a non-zero net spin current is established across the interface^23–25^. When the F layer is hotter than the N layer, a spin Seebeck current of magnons generated by the temperature gradient in the F layer flows towards the F/N interface and is converted at the interface into a pure spin current carried by conduction electrons in the N layer^22, 26–28^. When the N layer is hotter than the F layer, the net spin current across the F/N interface reverses its direction resulting in angular momentum injection from the N layer into the F layer^17^. In this process, fluctuating spins of the conduction electrons of the hotter N layer generate a net flow of magnons into the colder F layer^29, 30^, which increases the magnon density in the F layer above its equilibrium value^23–25, 30, 31^.

The net spin current injected into the F layer by a temperature gradient across the F/N interface was theoretically predicted^19, 23–25, 32, 33^ and experimentally demonstrated^17, 29, 34, 35^ to reduce the magnetic damping of the F layer magnetization. The action of such thermal spin current can be described in terms of an antidamping thermal spin torque^30^, which can be called spin Seebeck torque. However, full cancellation of the F layer damping by the temperature gradient needed for the excitation of magnetic auto-oscillations has not been achieved.

In the following, we demonstrate operation of an STO driven by pure spin current arising from a temperature gradient across an F/N interface. This device realizes a major goal of spin caloritronics—thermal energy harvesting for the manipulation of magnetization^33^.

## Results

### Sample description

**Fig. 1:**
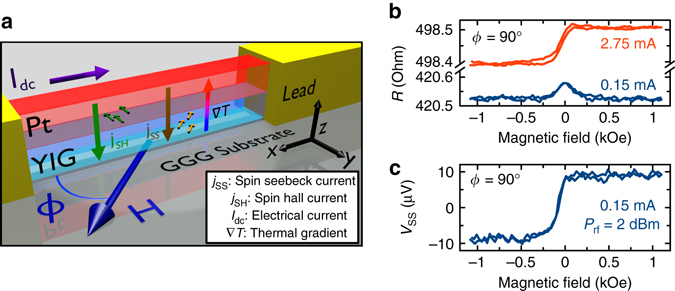
YIG/Pt nanowire magnetoresistance. **a** Sketch of the YIG/Pt nanowire spin torque oscillator. *Arrows* across the YIG/Pt interface illustrate the flow of spin Hall *j*_SH_ and spin Seebeck *j*_SS_ currents with corresponding smaller arrows representing the spin current polarization. The directions of the temperature gradient ∇*T* perpendicular to the YIG/Pt interface, the direct current *I*_dc_, and the magnetic field *H* are depicted by arrows. **b** Magnetoresistance *R* of the YIG/Pt nanowire measured at low (*I*_dc_ = 0.15 mA) and high (*I*_dc_ = 2.75 mA) direct current bias for a magnetic field applied in the sample plane at the field angle *ϕ* = 90° with respect to the wire axis. **c** Spin Seebeck voltage *V*_SS_ induced in the nanowire by a large microwave current (microwave power *P*_rf_ = 2 dBm) as a function of magnetic field

We study STOs based on pure spin current injection into a nanowire of an insulating ferrimagnet yttrium iron garnet Y_3_Fe_5_O_12_ (YIG). The 350 nm wide, 15 μm long nanowire devices are made by e-beam lithography and ion mill etching from Gd_3_Ga_5_O_12_(GGG substrate)/YIG(23 nm)/Pt(8 nm) films grown by sputter deposition^36^. Two Al(4 nm)/Pt(2 nm)/Cu(15 nm)/Pt(2 nm) leads with an inter-lead gap of 2.5 μm are attached to the ends of the nanowire for application of an electric current along the wire length as shown in Fig. 1a (and Supplementary Fig. 1). Since YIG is an insulator, the electric current and the associated ohmic heat generation are confined to the Pt layer of the wire, which results in a large temperature gradient ∇*T* in the YIG film perpendicular to the YIG/Pt interface at high current densities (Supplementary Note 1 and Supplementary Fig. 2).

### Magnetoresistance measurements

**Fig. 2:**
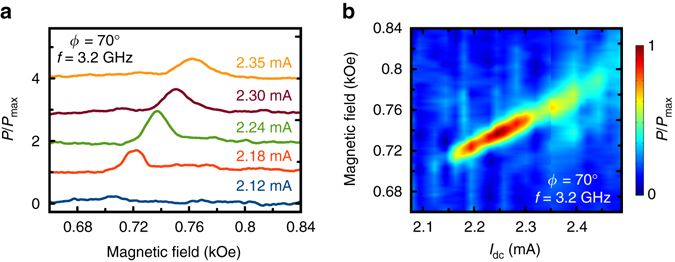
YIG/Pt nanowire microwave signal generation. **a** Spectra of normalized microwave power *P*/*P*_max_ generated by the nanowire at the frequency *f* = 3.2 GHz and magnetic field angle *ϕ* = 70° as a function of magnetic field at several direct current biases (vertically offset for clarity). **b** *Color* plot of microwave power generated by the nanowire at 3.2 GHz as a function of magnetic field and direct current bias

**Fig. 3:**
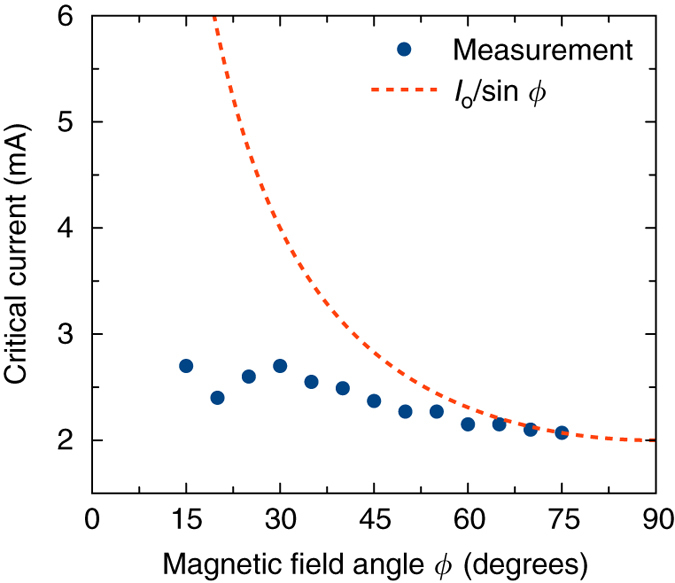
Angular dependence of the critical current. Critical current for the onset of auto-oscillations as a function of in-plane magnetic field direction *ϕ*. The line shows the expected behavior due to only a spin Hall current (with a fitting parameter *I*_0_) in the absence of a spin Seebeck current

**Fig. 4:**
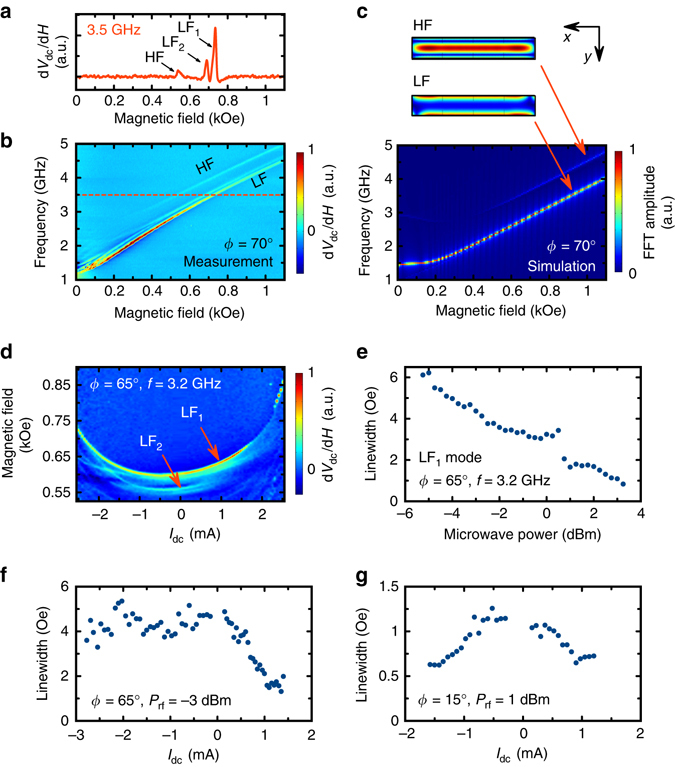
ST-FMR measurements. **a** A single spin torque ferromagnetic resonance spectrum measured at a microwave frequency *f* = 3.5 GHz and magnetic field angle *ϕ* = 70°. Low-frequency (LF) and high-frequency (HF) modes are observed. **b** ST-FMR spectra of the YIG/Pt nanowire measured as a function of magnetic field and drive frequency at the field angle *ϕ* = 70°. **c** Micromagnetic simulation of the spin wave eigenmode spectra in the YIG/Pt nanowire with a top view of the spatial dependence of the LF and HF mode amplitudes. **d** ST-FMR spectra measured as a function of magnetic field and direct current bias current *I*_dc_ for microwave power *P*_rf_ = −3 dBm. **e** Linewidth of the LF_1_ measured as a function of the microwave drive power *P*_rf_. **f** Linewidth of the LF_1_ mode as a function of direct current *I*_dc_ for *ϕ* = 65° and *P*_rf_ = −3 dBm. **g** Linewidth of the LF_1_ mode as a function of direct current for *ϕ* = 15° and *P*_rf_ = 1 dBm

Figure 1b shows the nanowire resistance as a function of magnetic field *H* applied in the plane of the sample, perpendicular to the wire axis (in-plane field angle *ϕ* = 90°). At a low direct bias current *I*_dc_ = 0.15 mA, we observe negative magnetoresistance (MR) that saturates when *H* exceeds the YIG nanowire’s magnetic shape anisotropy field (0.2 kOe). These data are well explained by spin Hall magnetoresistance (SMR) arising from spin Hall current in Pt flowing perpendicular to the YIG/Pt interface^37, 38^. At a higher current bias *I*_dc_ = 2.75 mA, the resistance of the wire saturates at different values for positive and negative *H*. This field-antisymmetric component of the MR arises from a large spin Seebeck current driven by ∇*T* and the inverse spin Hall effect in Pt^23, 39–46^. As illustrated in Fig. 1c, application of a large microwave current instead of *I*_dc_ also heats the Pt layer and results in a similar field-antisymmetric spin Seebeck voltage *V*_SS_ induced in the nanowire. This demonstrates that the field-antisymmetric MR in Fig. 1b arises from ∇*T* rather than direct current bias^23, 42^. All measurements presented in Figs 1–4 are made at the sample bath temperature of 140 K chosen such that the nanowire temperature is near room temperature at the highest bias current (3 mA) employed in our experiments (Methods, Supplementary Note 1 and Supplementary Fig. 3).

### Microwave generation

The MR data in Fig. 1b and c suggest that pure spin currents driven by both the spin Hall effect and ∇*T* can flow across the YIG/Pt interface when a direct electric current is applied to the Pt layer. As discussed in the introduction, these pure spin currents can apply antidamping spin torques to the magnetization of YIG^7, 18, 22, 30^. If the negative effective damping due to these spin currents exceeds the positive magnetic damping of the YIG nanowire, GHz-range persistent auto-oscillatory dynamics of the YIG magnetization can be excited. Owing to the YIG/Pt nanowire MR, these auto-oscillations give rise to the sample resistance oscillations *δR*_ac_ and a microwave voltage *V*_ac_ ~ *δR*_ac_ *I*_dc_ generated by the sample at the frequency of the auto-oscillations. We detect the auto-oscillations of magnetization via measurements of the microwave power spectral density $P \sim V_{{\rm{ac}}}^2$ generated by the sample under direct current bias.

In contrast to conventional measurements of the microwave power emitted by STO as a function of frequency at fixed *H* ^12^, we measure the emitted power at a fixed frequency (3.2 GHz in Fig. 2a and b) as a function of the applied magnetic field *H*. As discussed in the Supplementary Note 2, this STO characterization method greatly improves the signal-to-noise ratio in comparison to the conventional method and allows us to measure fW-scale microwave signals generated by nanomagnetic devices (Supplementary Fig. 4).

Our measurements of the microwave signal generated by the YIG/Pt nanowire reveal that full compensation of the YIG layer damping by the spin Hall and the thermal spin currents can be achieved, and auto-oscillations of the YIG magnetization can be excited. Figure 2a shows *P* generated by the sample at 3.2 GHz measured as a function of *H* applied at *ϕ* = 70° for several values of *I*_dc_. These data reveal a sharp onset of the microwave emission from the sample for *I*_dc_ exceeding the critical value of 2.15 mA. The peak of *P*(*H*) for *I*_dc_ = 2.15 mA is observed at *H* = 0.715 kOe, which is the resonance field of the lowest-frequency spin wave eigenmode of the YIG nanowire at this value of *I*_dc_, as shown in the next section.

Figure 2b demonstrates that the peak value of *P*(*H*) shifts to higher magnetic fields with increasing *I*_dc_. As discussed in the next section, this shift arises from reduction of the magnetization and magnetic shape anisotropy of the YIG wire caused by ohmic heating of the sample. The amplitude of the peak in *P*(*H*) first increases with increasing *I*_dc_ reaching the maximum value *P*_max_ ≈ 0.1 fW MHz^−1^ at *I*_dc_ = 2.25 mA and then gradually decreases reaching the background noise level at *I*_dc_ ≈ 2.5 mA for *ϕ* = 70°. The integrated microwave power generated by the sample at *I*_dc_ = 2.25 mA is estimated to be 6 fW (Supplementary Note 3).

The field-frequency relation of the spin wave mode and thus the auto-oscillation frequency can be efficiently controlled via changing the nanowire width and thereby modifying its magnetic shape anisotropy^47^. For the nanowire magnetized by a transverse magnetic field, the resonance field increases (the resonance frequency decreases) with decreasing nanowire width^47^, as confirmed by our measurements of a 90 nm wide YIG/Pt nanowire described in the Supplementary Note 2.

In order to gauge the relative contributions of the spin Hall and spin Seebeck currents to the excitation of the auto-oscillatory dynamics, we make measurements of the critical current for the onset of the auto-oscillations as a function of the in-plane magnetic field direction *ϕ*. In-plane rotation of the YIG magnetization changes its direction with respect to the polarization of the spin Hall current, which leads to a 1/sin *ϕ* dependence of the critical current when the spin Hall current is acting alone^7^. In contrast, the spin Seebeck current polarization is always collinear to the direction of the YIG magnetization. For ∇*T* shown in Fig. 1a, the polarization is antiparallel to the magnetization of YIG, resulting in an antidamping spin torque^22, 24, 25, 30^. The critical current due to the spin Seebeck current driving the magnetic precession is expected to be only weakly dependent on *ϕ* as long as the auto-oscillation frequency is fixed^31^ as in our measurements.

Figure 3 shows the angular dependence of the critical current measured for our YIG/Pt nanowire in the range of *ϕ* from 15 to 75°. For *ϕ* near 0° and 90°, the microwave power generated by the device is too small to be measured by our technique due to the weak angular dependence of MR near these angles. The measured angular dependence of the critical current is much weaker than the 1/sin *ϕ* dependence expected from the spin Hall current^7, 12^ suggesting a significant contribution to antidamping from the spin Seebeck current.

Current-driven auto-oscillations of magnetization were recently observed in microdisks of YIG/Pt bilayers at room temperature^7^. In these structures, we estimate the temperature gradient across the YIG film at the critical current (≈0.033 K nm^−1^) to be much weaker than in our devices (≈0.26 K nm^−1^). This is because the nanowire geometry in our device strongly enhances heat confinement within the Pt layer (Supplementary Note 1). The measured angular dependence of the critical current in YIG/Pt microdisks closely followed the 1/sin *ϕ* dependence^7^ indicating that the auto-oscillations of the YIG magnetization in these samples with small ∇*T* were driven purely by spin Hall torque. Comparison of these results to the data in Fig. 3 lends further support to a large *ϕ*-independent spin Seebeck antidamping in our YIG/Pt nanowire STOs. While quantitative fitting of the data in Fig. 3 is difficult because the temperature dependences of the spin Hall and spin Seebeck currents are not well understood, it is clear that auto-oscillations of magnetization in our YIG/Pt nanowires can be driven primarily by spin Seebeck current for a magnetic field direction near the nanowire axis.

### Spin torque ferromagnetic resonance measurements

To gain further understanding of the mechanisms leading to the excitation of the auto-oscillations, we measure the spectrum of spin wave eigenmodes of the nanowire by spin torque ferromagnetic resonance (ST-FMR)^48, 49^. In this method, a rectified voltage *V*_dc_ generated^50^ by the sample in response to the applied microwave current is measured as a function of the drive frequency and external magnetic field. Resonances in *V*_dc_ are observed at the frequency and field values corresponding to spin wave eigenmodes of the system^51^. To improve the sensitivity of the method, we modulate the applied magnetic field and measure $\frac{{{\rm{d}}{V_{{\rm{dc}}}}}}{{{\rm{d}}H}}$ ^52^. In Fig. 4a and b, two spin wave eigenmodes of the YIG nanowire are clearly seen in the ST-FMR spectra measured at *I*_dc_ = 0.

Patterning of the YIG film into the nanowire gives rise to lateral confinement of spin waves along the wire width, which results in quantization of the spin wave eigenmode spectrum^12, 47^. The resonances observed in ST-FMR spectra in Fig. 4a and b arise from the low-frequency sector of this quantized spin wave mode spectrum. Precise identification of the modes measured by the ST-FMR spectroscopy can be accomplished via comparison of the ST-FMR data in Fig. 4b to the spin wave eigenmode spectra obtained from micromagnetic simulations^53^ (Supplementary Note 4). Figure 4c shows the dependence of the simulated eigenmode frequencies on magnetic field for *ϕ* = 70°. Similar to the ST-FMR data in Fig. 4b, two eigenmodes are present in the simulations with their frequencies being close to those measured by ST-FMR. The spatial profiles of the amplitudes of these modes displayed in Fig. 4c for *ϕ* = 70° show that the high-frequency (HF) mode is a standing spin wave with two nodes in the direction perpendicular to the wire axis. For the low-frequency (LF) mode, the amplitude maxima lie near the wire edges^12^. The LF mode exhibits fine splitting (labeled as LF_1_ and LF_2_ in Fig. 2a), which arises from geometric confinement^54, 55^ of the mode along the wire length^12, 47^.

Figure 4d shows ST-FMR spectra of the wire measured at 3.2 GHz and *ϕ* = 65° as a function of the direct bias current. At currents below the critical, the resonance fields of the LF_1_ and LF_2_ modes exhibit a current-induced shift. This shift has a quadratic component from the reduction of the YIG magnetization by ohmic heating, as established by previous studies of magnetic nanodevices^55, 56^. The linear component of the frequency shift is due to the spin Hall current^57^. At the critical current for the onset of auto-oscillations (2.2 mA), the LF_1_ mode resonance field is equal to the field at which auto-oscillations of magnetization are observed for this measurement frequency and direction of *H*. This demonstrates that the observed auto-oscillatory dynamics arises from the LF_1_ mode.

Quantitative measurements of the spin wave mode intrinsic linewidth (Supplementary Note 5) by ST-FMR in our samples present significant difficulties as illustrated in Fig. 4e, which shows the linewidth of the LF_1_ mode as a function of microwave drive power *P*_rf_. The observed linewidth decrease with increasing *P*_rf_ cannot be explained by a nonlinear lineshape distortion that is known to increase the linewidth with increasing power^48^. However, it is consistent with the antidamping action of the spin Seebeck current arising from microwave-induced heating of the Pt layer. Much lower values of the drive power needed for measuring the intrinsic linewidth (i.e. free from both spin Seebeck and nonlinear contributions) do not produce a measurable ST-FMR signal due to the small MR of the samples (Fig. 1b). Therefore, ST-FMR measurements of the linewidth presented below are invasive. Nevertheless they give important qualitative information on the relative contributions of the spin Hall and spin Seebeck currents to the current-induced antidamping torque.

## Discussion

The linewidth of the spin wave eigenmodes below the critical current in a spin Hall oscillator decreases with *I*_dc_ for one (antidamping) current polarity and increases for the other (damping) polarity because the damping-like component of the spin Hall torque is linear in *I*_dc_^7, 16, 55, 56^. The dependence of the LF_1_ mode linewidth on *I*_dc_ in our YIG/Pt devices exhibits a radically different behavior illustrated in Fig. 4f. The linewidth measured at *P*_rf_ = −3 dBm decreases for *I*_dc_ > 0 but remains nearly constant for *I*_dc_ < 0. These data can be explained via a significant antidamping spin Seebeck torque^23, 30^ driven by ∇*T*. Indeed, both direct current polarities give rise to the same degree of ohmic heating in Pt and therefore to the same spin Seebeck current acting as antidamping^23–25, 29, 34, 35^. The rapid decrease of the linewidth with positive current at *ϕ* = 65° shown in Fig. 4f is due to the combined action of the antidamping torques from spin Hall and spin Seebeck currents. At a negative current bias, the spin Hall torque acts as positive damping while the spin Seebeck torque acts as antidamping. The competition between these two torques results in a weak variation of the linewidth with current for *I*_dc_ < 0 and *ϕ* = 65°.

Application of the magnetic field nearly parallel to the nanowire axis (*ϕ* = 15°) allows us to further separate the action of the spin Seebeck torque from that of the spin Hall torque. In this configuration, the polarization of the spin Hall current and magnetization are nearly perpendicular, resulting in a negligibly small damping/antidamping spin Hall torque^11^. Thus, the *ϕ*-independent antidamping torque from the spin Seebeck current^23, 30, 34, 35^ is expected to dominate the magnetization dynamics. ST-FMR measurement of the LF_1_ mode linewidth as a function of current bias is shown in Fig. 4g. For this measurement we must employ a higher microwave drive (*P*_rf_ = 1 dBm) due to the reduced ST-FMR signal in a nanowire magnetized close to its axis. The data illustrate that we indeed observe the linewidth decrease with increasing current bias for both current polarities, as expected for the dominant antidamping from the spin Seebeck current and negligible damping/antidamping from the spin Hall current. The linewidth decrease with increasing current bias in Fig. 4f and g cannot be explained by a temperature dependence of damping^58^, since we find the linewidth to be nearly constant in the temperature range from 140 to 300 K (Supplementary Note 6 and Supplementary Fig. 5).

A microscopic mechanism of the antidamping action of the spin Seebeck current in F/N bilayers was theoretically discussed in ref. ^30^. In this mechanism, a spin Seebeck current driven from the N into the F layer by ∇*T* generates a non-equilibrium population of incoherent magnons in the F layer. Nonlinear magnon scattering transfers angular momentum from this incoherent magnon cloud to a low-frequency coherent spin wave mode and thereby reduces the effective damping of the mode. In this picture, the auto-oscillatory spin wave dynamics above the critical current can be viewed as bosonic condensation^59^ of non-equilibrium incoherent magnons generated by a thermal spin current into a coherent low-frequency spin wave mode^19, 60^. While the order of magnitude of the threshold thermal bias measured in our experiment is reasonable, according to the theory (where the threshold temperature difference between magnons in YIG and electrons in Pt is set by the frequency of the auto-oscillating mode), the quantitative details depend on the interplay of the magnon–magnon, magnon–phonon, and magnon–electron scatterings. Future efforts are called upon to clarify the relative importance of these in YIG/Pt heterostructures.

In conclusion, we observe current-driven auto-oscillations of the magnetization in YIG/Pt bilayer nanowires. Measurements of the angular and current bias dependence of antidamping spin torques in this system reveal that the auto-oscillatory dynamics are excited by a combination of spin Hall and spin Seebeck currents. We show that the spin Seebeck current resulting from ohmic heating of Pt can be the dominant drive of auto-oscillations of the YIG magnetization. Our measurements demonstrate that ohmic heating can be utilized for generation of tunable microwave signals and coherent spin waves. While the output power of the YIG/Pt STO studied here is low, we expect that the spin Seebeck drive mechanism demonstrated here can be utilized in other types of STOs with higher magnetoresistance and output microwave power. Our results pave the way towards spin caloritronic devices based on ohmic heat harvesting.

## Methods

### Experimental technique

All measurements reported in this study are performed in a continuous flow helium cryostat, where the sample is surrounded by a helium gas injected into the sample space via a needle valve at the bottom of the sample space. The temperature of the helium gas can be controlled in the range from 4.2 to 300 K via a feedback loop using a heater and a thermometer near the needle valve. The sample is attached to a custom made brass sample holder placed near the helium gas injection port, and the contact pads of the YIG/Pt nanowire device are electrically connected to a short coplanar waveguide (CPW) via 4 mm long aluminum wire bonds. The brass sample holder is electrically connected to the ground of the CPW, and the central conductor of the CPW is soldered to a microwave K-connector. The K-connector of the sample holder is connected to the microwave electronics outside of the cryostat (Supplementary Fig. 4a) via a 1 m long cryogenic microwave cable. The frequency-dependent microwave signal attenuation/amplification of the entire microwave circuit from the sample holder to the spectrum analyzer is characterized using a microwave network analyzer, and the reported power levels are corrected for this loss/amplification. An electromagnet on a rotating stage placed outside the cryostat allows for application of a magnetic field up to 4 kOe at an arbitrary direction within the sample plane.

The microwave signal generated by the YIG/Pt nanowire oscillator is low-level (power spectral density below 1 fW MHz^−1^) because magnetoresistance of the sample is small. In order to reliably measure the spectral properties of such low-level signals, we develop an ultra-sensitive technique for detection of microwave signals generated by spin torque oscillators (STOs). This technique improves the signal-to-noise ratio over the conventional technique used for measurements of STO microwave emission spectra by two orders of magnitude. The key feature of this technique is the harmonic modulation of the applied magnetic field and lock-in detection of the emitted microwave power at the modulation frequency, which greatly diminishes the influence of non-magnetic noise on the measured signal (Supplementary Note 2).

### Data availability

All data supporting the findings of this study are available within the article and the Supplementary Information file, or are available from the corresponding author on reasonable request.

## Electronic supplementary material

Supplementary Information
