# Supplementary material for: Spin caloritronic nano-oscillator
Source: Nat Commun. 2017 Jul 18;8:117. doi: 10.1038/s41467-017-00184-5 (PMC5527023; doi:10.1038/s41467-017-00184-5)
Supplement: Supplementary file 1 — Supplementary Information [file 41467_2017_184_MOESM1_ESM.pdf]

File Name: Supplementary Information
Descriptions: Supplementary Figures, Supplementary Notes and Supplementary References

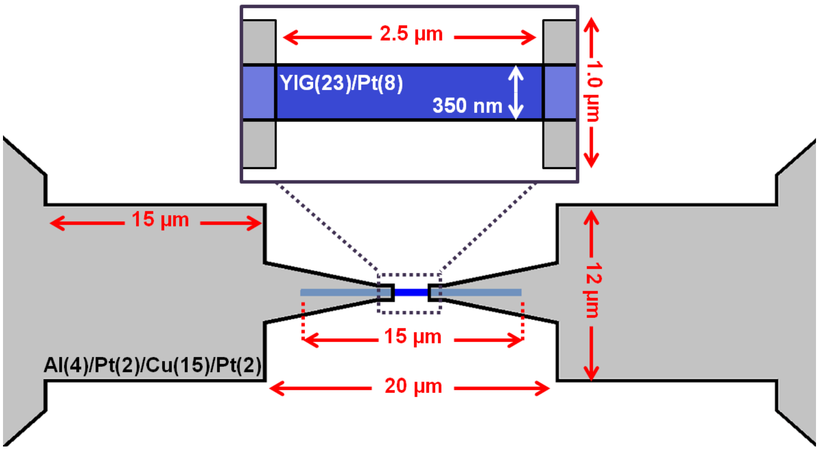

Supplementary Figure 1: **Nanowire device sketch.** Sketch of the YIG/Pt bilayer nanowire device with two Al/Pt/Cu/Pt leads. The nanowire depicted in blue color is partially covered by the electrical leads depicted in grey. The expanded view panel shows the active region of the nanowire. Lateral dimensions as well as nanowire and lead material stacks including layer thicknesses in nanometers are shown.

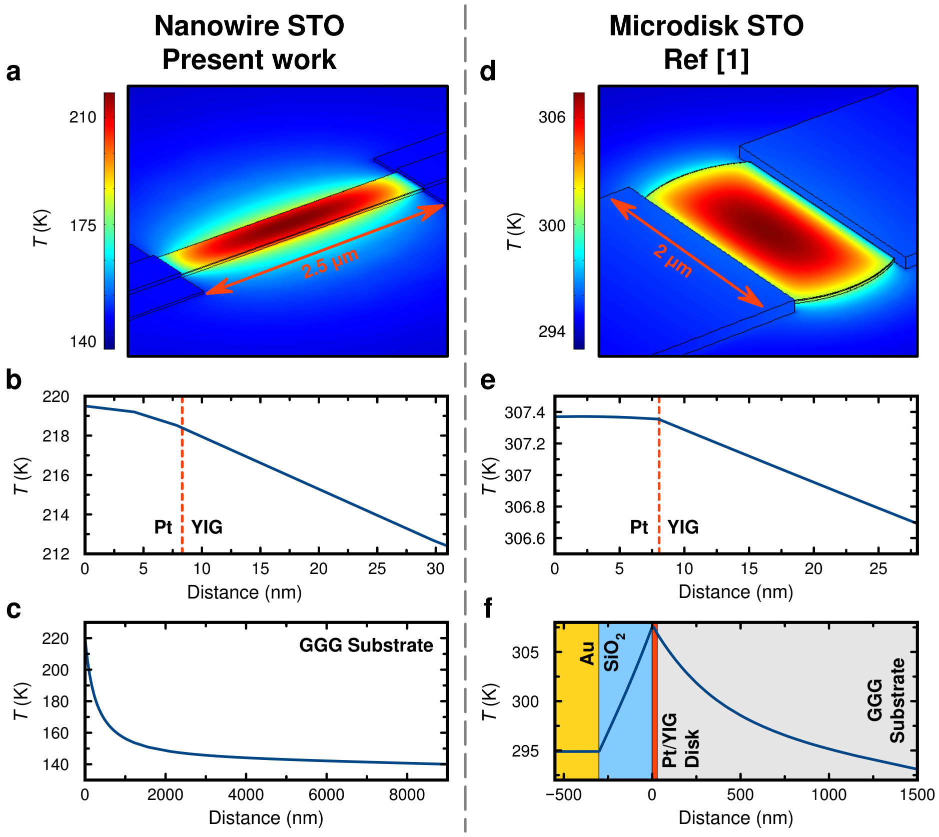

Supplementary Figure 2: **Ohmic heating and temperature gradients.** **a** COMSOL simulation of temperature distribution due to ohmic heating in a YIG(23 nm)/Pt(8 nm) nanowire spin torque oscillator (STO) at $I_{dc} = 2.5\,mA$ and 140 K bath temperature. **b** Temperature depth profile in the center of the nanowire. **c** Temperature depth profile within the GGG substrate under the nanowire center. **d** COMSOL simulation of temperature distribution due to ohmic heating in a YIG(20 nm)/Pt(8 nm) microdisk studied in Ref. 1 at $I_{dc} = 7.4\,mA$ and 293 K bath temperature. **e** Temperature depth profile in the center of the microdisk. **f** Temperature depth profile in the SiO_2/Au overlayer above the microdisk and in the GGG substrate below the microdisk center.

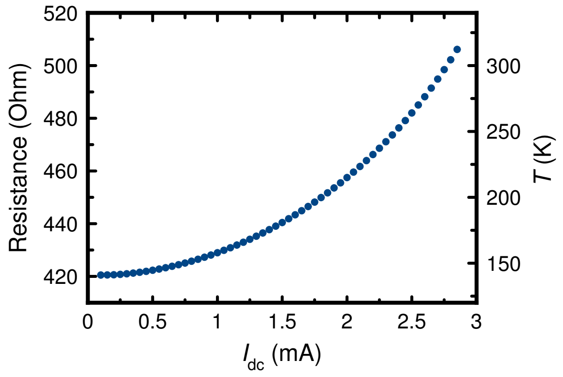

Supplementary Figure 3: **Pt nanowire resistance as a function of direct current bias.** The right ordinate axis shows the corresponding wire temperature that was obtained from measurements of the wire resistance as a function of bath temperature at small bias current.

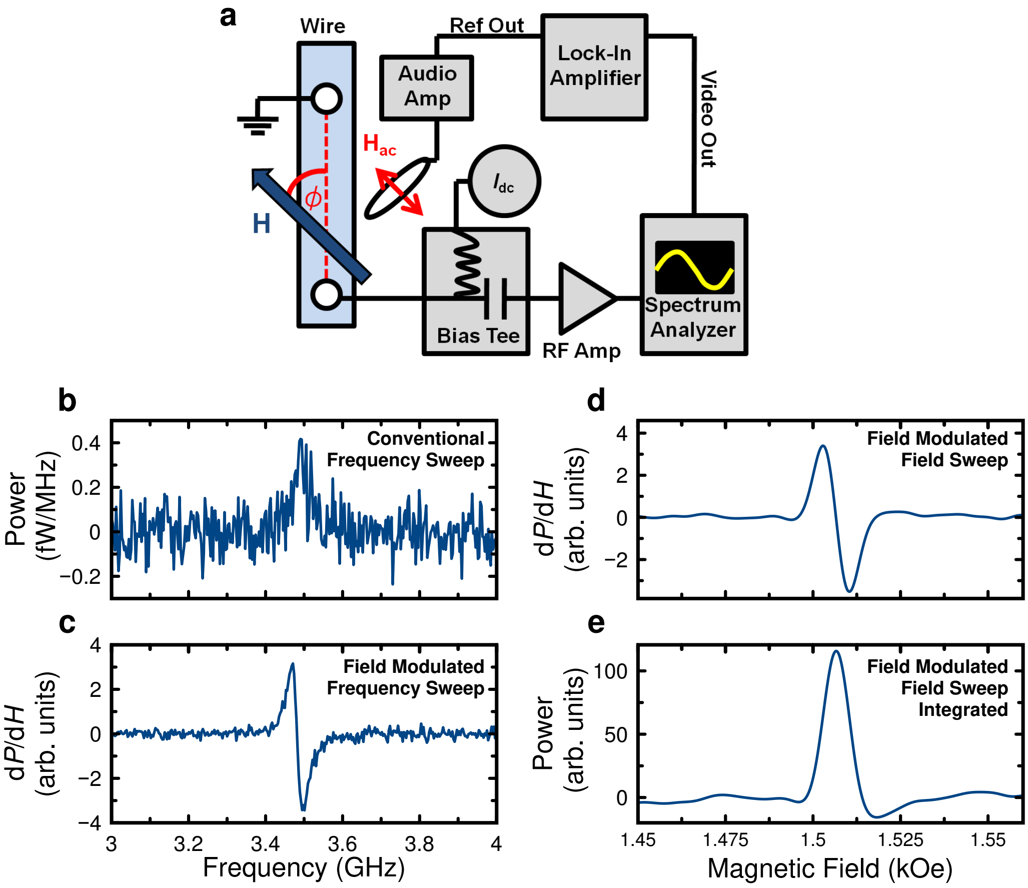

Supplementary Figure 4: **Microwave emission measurements.** **a** Schematic of the experimental setup for measurements of spin torque oscillator (STO) microwave emission with magnetic field modulation. The nanowire with magnetic field H applied at an angle ϕ is supplied with a direct current I_{dc} via a bias tee. The microwave signal is amplified and detected using a spectrum analyzer. Its video-out signal is processed with a lock-in amplifier. The reference-out signal is used to generate a modulation field H_{ac} using an audio amplifier. **b-e** Microwave emission spectra from a 90 nm wide YIG(23 nm)/Pt(8 nm) nanowire STO with a 0.9 μm long active region measured at H near 1.5 kOe, $\phi = 70°$ and $I_{dc} = 1$ mA by different techniques. **b** Conventional technique without field modulation. **c** Field modulation technique at fixed magnetic field and swept frequency. **d** Field modulation technique at fixed detection frequency and swept magnetic field. **e** Spectrum from **d** integrated with respect to the magnetic field.

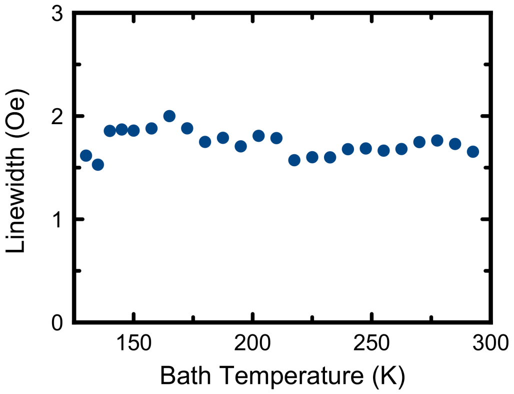

Supplementary Figure 5: **Temperature dependence of linewidth.** ST-FMR linewidth of the LF_1 mode as a function of bath temperature measured at the field angle $\phi = 15°$, drive frequency of 3.2 GHz and drive power $P_{rf} = 1$ dBm.

Supplementary Note 1. Ohmic Heating.

In order to estimate the magnitude of thermal gradients arising from ohmic heating, we carried out finite element simulations of coupled electrical and thermal transport in the YIG/Pt nanowire devices using COMSOL Multiphysics package [2]. We employed a fully realistic three-dimensional nanowire device geometry illustrated in Supplementary Figures 1 and 2a. A detailed sketch of the YIG/Pt nanowire device with two electrical leads used in the experiment and simulations is shown in Supplementary Figure 1. The device consists of a 350 nm wide 15 μm long YIG(23 nm)/Pt(8 nm) nanowire with two Al(4 nm)/Pt(2 nm)/Cu(15 nm)/Pt(2 nm) electrical leads attached to the wire ends as shown in Supplementary Figure 1. The leads are separated by a 2.5 μm gap that defines the active region of the nanowire STO device.

In these simulations, we used the temperature-dependent heat conductivities and heat capacities of YIG and GGG as reported in Ref. 3. The Pt layer resistivity in our YIG(23 nm)/Pt(8 nm) nanowire devices was measured in the temperature range from 140 K to 300 K and found to be linear as expected: $\rho(T) = \rho_0(1 + \alpha T)$ with $\rho_0 = 3.35 \times 10^{-7}\Omega\cdot$m and $\alpha = 1.59 \times 10^{-3}$K^{-1}, which is similar to previously reported values in thin Pt films [4, 5]. The temperature-dependent heat capacity of Pt reported in Ref. 6 was employed in the simulations, and the thermal conductivity of the Pt layer was calculated from its electrical conductivity via the Wiedemann-Franz law. Literature values of the thermal and electrical conductivity and heat capacity of the lead materials were employed [2].

Supplementary Figure 2a shows the calculated spatial distribution of temperature in the YIG/Pt nanowire device studied in this work at the bath temperature of 140 K and direct current bias $I_{dc} = 2.5$ mA that is similar to the critical current. Supplementary Figures 2b and 2c show the depth profiles of the temperature in the center of the YIG/Pt wire. These figures reveal that the Pt layer temperature rises to 220 K. The temperature in the YIG layer rapidly decreases with depth resulting in a large temperature gradient $\nabla T = 0.26$ K nm^{-1} across the YIG layer thickness. This high degree of ohmic heating and the large value of ∇T result from the high resistivity of the Pt layer and efficient heat channeling into the YIG underlayer in the nanowire geometry employed in our experiment. In this geometry, the metallic leads do not function as efficient heat sinks because their overlap area with the nanowire is relatively small, which results in a high degree of ohmic heating of the Pt nanowire and dissipation of this heat is mainly through the GGG/YIG underlayers. The quasi-one-dimensional nature of the Pt nanowire heat source and the three-dimensional character of the heat flow in the GGG substrate further enhance ∇T across the thickness of the YIG layer.

The validity of these COMSOL simulations can be directly checked against the experiment because the temperature of the Pt wire can be determined by measuring its resistance. Supplementary Figure 3 shows the resistance of the Pt nanowire measured as a function of direct current bias. These data and the linear relation between the Pt nanowire resistance and temperature reveal that the Pt nanowire temperature at the bath temperature of 140 K and $I_{dc} =$ 2.5 mA is 260 K. We therefore conclude that the COMSOL simulations underestimate the degree of ohmic heating of the Pt wire and that the actual temperature gradient across the YIG film thickness is likely to exceed that predicted by the simulations.

We also employed COMSOL simulations to evaluate the ohmic heating in the YIG/Pt microdisk spin torque oscillators investigated in Ref. 1. The microdisk device geometry used in our simulations and experimentally studied in Ref. 1 is shown in Supplementary Figure 2d. The system consists of a 2 μm-diameter YIG(20 nm)/Pt(8 nm) disk

defined on top of a GGG substrate. Two Ti(20 nm)/Au(80 nm) leads are attached to the disk with the inter-lead gap of 1 μm. The system is covered with an SiO_2(300 nm)/Au(250 nm) bilayer (not shown in Supplementary Figure 2d). The Pt layer resistivity $\rho = 1.7 \times 10^{-7}\Omega\cdot$m was directly measured for this system [1], and thermal conductivity of Pt was calculated via the Wiedemann-Franz law. In these simulations we also use the experimental parameters of Ref. 1: a bath temperature of 293 K and a critical current of 7.4 mA. Literature values of the temperature-dependent electrical conductivity, thermal conductivity and heat capacity of Au, Ti, SiO_2, YIG and GGG [2, 3, 6] were employed in the simulations.

Supplementary Figures 2e,f show the depth dependence of the temperature in the center of the disk at $I_{dc} = 7.4$ mA. It is clear from these figures that ohmic heating of the Pt layer is substantially smaller than in our nanowire devices due to the lower electrical resistivity of the Pt layer employed in Ref. 1 and better heat sinking by the Ti/Au leads having significant contact area with the microdisk. Combined with this, these devices are encased by 300 nm of silicon oxide to allow electrical isolation from a 250 nm thick Au antenna, providing further heat sinking. The resulting temperature gradient in the YIG film across its thickness at the critical current is only 0.033 K nm^{-1} – an order of magnitude smaller than that in our nanowire devices. Therefore, it is not surprising that the antidamping torque in these devices predominantly arises from spin Hall current with a negligible contribution from spin Seebeck current as evidenced by the $1/\sin\phi$ angular dependence of the critical current observed for these samples [1].

Supplementary note 2. Field modulated detection of microwave emission.

Supplementary Figure 4a schematically illustrates the experimental setup employed in our field-modulated microwave emission measurements. A low-frequency (∼1 kHz), small-amplitude harmonic magnetic field H_{ac} is applied to an STO sample parallel to a constant external magnetic field H_{dc}. A direct current bias I_{dc} is supplied from a custom built low noise current source and applied to the STO via a Picosecond 5541A-104 bias tee. The direct bias current excites the self-oscillations of magnetization. The microwave signal emitted by the sample is then amplified through a Miteq AMF-6F-00100400-10-10P low noise microwave amplifier with 62 dB gain, noise figure of 1 dB, and frequency band of 0.1–4 GHz. The amplified signal is then sent to an Agilent E4408B spectrum analyzer, configured in a single-frequency continuous detection mode. This configuration measures the integrated microwave power in a 5 MHz bandwidth around a fixed measurement frequency. The STO generation frequency is modulated by H_{ac}, which results in a modulation of the STO power at the measurement frequency. The modulated STO emission power $\frac{dP}{dH}$ is measured by a Signal Recovery 7225 lock-in amplifier via a video output port of the spectrum analyzer. In order to obtain a field-modulated STO emission spectrum, the data is collected point-by-point by stepping the measurement frequency of the spectrum analyzer over a desired frequency range.

The conventional method of measuring STO microwave emission spectrum, in which the emission power is simply recorded as a function of frequency, did not yield a signal exceeding the noise floor for the 350 nm wide YIG/Pt nanowire samples discussed, and our field modulation technique was required to observe the signal. In order to quantitatively compare the field-modulated emission method to the conventional method, we employ an STO sample based on a 90 nm wide YIG(23 nm)/Pt(8 nm) nanowire with a 0.9 μm long active region. This STO generates higher microwave signals, which can be measured by the conventional technique as illustrated in Supplementary Figure 4b.

Supplementary Figures 4b,c directly compare the microwave emission spectra for conventional and field-modulated detection measured under identical conditions ($H = 1.5$ kOe, $\phi = 70°$, $I_{dc} = 1$ mA, measurement time 17 minutes). The conventional method gives a spectral peak with integrated power of 21 fW. In contrast, the field modulation method yields a prominent $\frac{dP}{dH}$ signal with high signal-to-noise ratio and the line shape similar to a Lorentzian curve derivative as illustrated in Supplementary Figure 4c.

Supplementary Figures 4d,e illustrate that the field modulation method can be further improved by sweeping external magnetic field instead of stepping the center-frequency as done in Supplementary Figure 4c. In Supplementary Figure 4d, the field-modulated emission signal is measured as a function of applied field giving the expected antisymmetric line shape. This signal can be directly integrated in magnetic field yielding a symmetric emission curve as a function of magnetic field. The development of this microwave detection technique increases the signal-to-noise ratio by two orders of magnitude, allowing detection of ultra low-level microwave signals emitted by magnetic devices.

Approximate calibration of the power scale for the field-integrated spectra such as that shown in Supplementary Figure 4e can be performed via comparison of the spectral peak amplitudes in Supplementary Figures 4b,e. We estimate the maximum power spectral density P_{max} generated by the 350 nm wide YIG/Pt nanowire shown in Fig. 2b to be approximately 0.1 fW MHz^{-1} and the corresponding integrated power to be approximately 6 fW.

We also note that the nanowire geometry can be used to tune both the frequency and the amplitude of the microwave signal generated by the YIG/Pt nanowire STO. We find that decreasing the width of the nanowire from 350 nm to

90 nm results in a decrease of the generated signal frequency. At the same time, the output power of the STO increases by over a factor of three. The decrease of the resonance frequency results from a higher demagnetization field in the narrower nanowire [7] while the increase of the output power can be attributed to a larger volume fraction of the wire occupied by the spin wave mode (see Fig. 4c).

Supplementary Note 3. Auto-oscillation amplitude.

The precession cone angle of the auto-oscillatory YIG magnetization can be estimated from the output microwave power of the YIG/Pt nanowire STO. The integrated microwave power $P_{\rm int}$ generated by an STO is proportional to the square of the direct current bias $I_{\rm dc}$ and the amplitude of resistance auto-oscillations $\delta R_{\rm ac}$ [8, 9]:

$$P_{\rm int} = \frac{1}{2R_{50}}\left(I_{dc}\delta R_{ac}\frac{R_{50}}{R+R_{50}}\right)^2 \tag{1}$$

where R is the sample resistance and R_{50} is the $50\,\Omega$ microwave transmission line impedance. Assuming the angular dependence of the YIG/Pt nanowire resistance is $R = R_0 + \frac{\Delta R}{2}\cos 2\phi$ as expected for SMR, the small-amplitude dynamic resistance oscillations $\delta R_{\rm ac}$ are related to the in-plane precession cone angle $\phi_{\rm c}$ in the macrospin approximation as:

$$\delta R_{ac} = \phi_c \Delta R \sin 2\phi_0 \tag{2}$$

where ϕ_0 is the equilibrium direction of the YIG magnetization. The maximum value of $\phi_{\rm c}$ achieved by the YIG magnetization in the 350 nm wide nanowire device can be calculated from Equations 1 and 2 by using the generated integrated power $P_{\rm int} = 6\,{\rm fW}$ and $\Delta R = 0.05\,\Omega$ extracted from Fig. 1b. This calculation gives the precession cone angle in the macrospin approximation $\phi_{\rm c} \approx 6^\circ$. Taking into account that the excited LF mode has the edge character as shown in Fig. 4c, and that the edge mode occupies approximately one third of the nanowire volume as predicted by our micromagnetic simulations, the amplitude of the YIG magnetization oscillations at the nanowire edge is estimated to be approximately 20°. We stress that this is merely an estimate because contributions to the generated microwave signal beyond SMR such as inductive signal generated by precessing magnetization [1] can be non-negligible in our nanowire devices.

Supplementary Note 4. Micromagnetic simulations

Micromagnetic simulations of the spin wave eigenmode frequencies of the YIG/Pt nanowires were performed using a modified version of the finite-differences simulation code MuMax^3 [10]. The nanowire was discretized into $2048 \times 32 \times 4$ cells, resulting in a cell size of $6.30 \times 8.75 \times 7.50\,{\rm nm}^3$. The saturation magnetization $M_{\rm s} = 130\,{\rm kA\,m^{-1}}$ [11] and the exchange constant $A_{\rm ex} = 3.5\,{\rm pJ\,m^{-1}}$ were used [12]. The spin wave eigenfrequencies were determined as the peak position of the Fourier-transform of the dynamic magnetization excited by a sinc-shaped magnetic field pulse [13]. The simulation time was chosen to be 25 ns, which results in an FFT frequency resolution of 40 MHz. The spin wave profiles shown in Fig. 4c are represented by the cell-specific Fourier amplitude.

Supplementary Note 5. Magnetic damping.

We use the conventional broadband FMR technique [14] to measure the FMR linewidth ΔH (defined as half width at half maximum of the Lorentzian absorption curve) as a function of frequency f for YIG(23 nm) and YIG(23 nm)/Pt(8 nm) films. The damping constant α and the inhomogeneous broadening parameter ΔH_0 are determined from the slope and zero-frequency intercept of the $\Delta H(f)$ data [15]. These measurements give $\alpha = 0.0014$ and $\Delta H_0 = 1.1\,{\rm Oe}$ for the YIG film and $\alpha = 0.0035$ and $\Delta H_0 = 2.6\,{\rm Oe}$ for the YIG/Pt bilayer. The linewidth ΔH measured at 3.2 GHz for the YIG/Pt bilayer film is found to be 6.7 Oe, which is similar to $\Delta H = 6\,{\rm Oe}$ at 3.2 GHz measured in the YIG/Pt nanowire device by ST-FMR at the lowest microwave power value (-5 dBm) as shown in Fig. 4e. This demonstrates that patterning of the YIG/Pt film into the nanowire device does not significantly alter the YIG layer damping.

Supplementary Note 6. Temperature dependence of linewidth.

Ohmic heating of Pt can affect magnetization dynamics in the YIG/Pt nanowire via both the thermal gradient across the YIG/Pt interface and the increase of the average temperature of YIG. To separate these effects, we carried out ST-FMR measurements as a function of bath temperature at a constant low direct current bias (0.15 mA). Supplementary Figure 5 shows temperature dependence of ST-FMR linewidth of the LF_1 mode measured at $\phi = 15^\circ$ in a YIG(23 nm)/Pt(8 nm) nanowire device nominally identical to that shown in Fig. 4. The measurements reveal that the linewidth is nearly constant in the 140 K − 300 K temperature range. The data in Supplementary Figure 5 demonstrate that the current-induced linewidth variation in Fig. 4g arises from spin Seebeck current driven by ∇T and not from temperature dependence of the YIG nanowire damping.

Supplementary References

[1] Collet, M. *et al.* Generation of coherent spin-wave modes in yttrium iron garnet microdiscs by spin-orbit torque. *Nat. Commun.* **7**, 10377 (2016).
[2] COMSOL Multiphysics v. 4.3a. www.comsol.com. COMSOL AB, Stockholm, Sweden.
[3] Slack, G. A. & Oliver, D. W. Thermal conductivity of garnets and phonon scattering by rare-earth ions. *Phys. Rev. B* **4**, 592–609 (1971).
[4] Zhang, X. *et al.* Experimental studies on thermal and electrical properties of platinum nanofilms. *Chin. Phys. Lett.* **23**, 936–938 (2006).
[5] Warkusz, F. The size effect and the temperature coefficient of resistance in thin films. *J. Phys. D: Appl. Phys.* **11**, 689–694 (1978).
[6] Furukawa, G. T., Reilly, M. L. & Gallagher, J. S. Critical analysis of heatcapacity data and evaluation of thermodynamic properties of ruthenium, rhodium, palladium, iridium, and platinum from 0 to 300 K. A survey of the literature data on osmium. *J. Phys. Chem. Ref. Data* **3**, 163–209 (1974).
[7] Duan, Z. *et al.* Spin wave eigenmodes in transversely magnetized thin film ferromagnetic wires. *Phys. Rev. B* **92**, 104424 (2015).
[8] Kiselev, S. I. *et al.* Microwave oscillations of a nanomagnet driven by a spin-polarized current. *Nature* **425**, 380–383 (2003).
[9] Duan, Z. *et al.* Nanowire spin torque oscillator driven by spin orbit torques. *Nat. Commun.* **5**, 5616 (2014).
[10] Vansteenkiste, A. *et al.* The design and verification of MuMax3. *AIP Adv.* **4**, 107133 (2014).
[11] Solt, I. H. Temperature dependence of YIG magnetization. *J. Appl. Phys.* **33**, 1189–1191 (1962).
[12] Klingler, S. *et al.* Measurements of the exchange stiffness of YIG films using broadband ferromagnetic resonance techniques. *J. Phys. D: Appl. Phys.* **48**, 015001 (2014).
[13] Venkat, G. *et al.* Proposal for a standard micromagnetic problem: Spin wave dispersion in a magnonic waveguide. *IEEE Trans. Magn.* **49**, 524–529 (2013).
[14] Kalarickal, S. S. *et al.* Ferromagnetic resonance linewidth in metallic thin films: Comparison of measurement methods. *J. Appl. Phys.* **99**, 093909 (2006).
[15] Farle, M. Ferromagnetic resonance of ultrathin metallic layers. *Rep. Prog. Phys.* **61**, 755–826 (1998).
